# Draft Genome Sequence of *Staphylococcus cohnii* subsp. *urealyticus* Isolated from a Healthy Dog

**DOI:** 10.1128/genomeA.01628-16

**Published:** 2017-02-16

**Authors:** David C. Bean, Sarah M. Wigmore, David W. Wareham

**Affiliations:** a Faculty of Science and Technology, Federation University Australia, Ballarat, Victoria, Australia; b Antimicrobial Research Group, Blizard Institute, Barts and The London School of Medicine and Dentistry, Queen Mary University London, London, United Kingdom

## Abstract

*Staphylococcus cohnii* subsp. *urealyticus* strain SW120 was isolated from the ear swab of a healthy dog. The isolate is resistant to methicillin and fusidic acid. The SW120 draft genome is 2,805,064 bp and contains 2,667 coding sequences, including 58 tRNAs and nine complete rRNA coding regions.

## GENOME ANNOUNCEMENT

As part of our effort to elucidate antibiotic resistance mechanisms among *Staphylococci* species recovered from companion animals, *S. cohnii* subsp. *urealyticus* strain SW120 was isolated from an ear swab of a 2-year-old female Weimaraner living in Ballarat, Australia. *S. cohnii* subsp. *urealyticus* was first described in 1991 (1) and methicillin-resistant strains have been recovered previously from companion animals (2, 3).

The isolate was recovered after enriching an ear swab in tryptic soya broth supplemented with 8% NaCl and incubated at 37°C for 24 h before subculture to Baird-Parker agar at 37°C for 48 h. The isolate was coagulase-negative in rabbit plasma, DNase-negative, and novobiocin-resistant. Sequencing of the 16SrRNA gene was unable to conclusively identify the isolate: this is consistent with previous reports that 16SrRNA sequencing has limited discriminatory ability among *Staphylococci* species (4). Instead, sequencing of housekeeping genes, including *cpn60* (5), *dnaJ* (4), *rpoB* (6, 7), and *tuf* (8) are preferred for speciation and in each case identified the isolate as *S. cohnii* subsp. *urealyticus* at ≥98% sequence identity. This was further confirmed by biochemical testing, in particular, the prolific hydrolysis of urea.

Sequencing was performed on the Illumina MiSeq platform (Illumina, Inc., San Diego, CA, USA). The closest available reference genome was identified using Kraken, and the reads were mapped to this using the Burrows–Wheeler aligner “mem” (BWA-mem) algorithm version 2 to assess the quality of the data. A *de novo* assembly of the reads was performed using SPAdes version 3.7.1, and the reads were again mapped back to the resultant contigs using BWA-mem. The assembly comprised 24 contigs (>1,000 bp) with a total length of 2,805,064 bp and a G+C content of 32.43% and an *N*_50_ value of 661,385 bp. Annotation was performed by the NCBI Prokaryote Genome Annotation Pipeline (PGAP) which showed a total of 2,667 coding sequences, including at least 58 tRNAs, nine complete rRNAs, and two CRISPR arrays.

ResFinder version 2.1 (9) failed to find any resistance determinants despite the isolate being phenotypically resistant to methicillin (by oxacillin and cefoxitin disk diffusion) and fusidic acid (MIC 4 µg/mL). Subsequent analysis of the genome identified a putative 238-residue protein with 100% identity to a putative *mecA* gene from an *S. cohnii* subsp. *cohnii* isolate. Fusidic acid resistance was imparted by the recently described *fusF* gene (10).

### Accession number(s).

This whole-genome shotgun project has been deposited at DDBJ/ENA/GenBank under the accession number MPPU00000000. The version described in this paper is the first version, MPPU01000000.

## References

[1] Kloos WE, Wolfshohl JF 1991 *Staphylococcus cohnii* subspecies: *Staphylococcus cohnii* subsp. *cohnii* subsp. nov. and *Staphylococcus cohnii* subsp. *urealyticum* subsp. nov. Int J Syst Bacteriol 41:284–289. doi:10.1099/00207713-41-2-284. 1854641

[2] Quitoco IM, Ramundo MS, Silva-Carvalho MC, Souza RR, Beltrame CO, de Oliveira TF, Araújo R, Del Peloso PF, Coelho LR, Figueiredo AM 2013 First report in South America of companion animal colonization by the USA1100 clone of community-acquired meticillin-resistant *Staphylococcus aureus* (ST30) and by the European clone of methicillin-resistant *Staphylococcus* *pseudintermedius* (ST71). BMC Res Notes 6:336. doi:10.1186/1756-0500-6-336. 23981343 PMC3765899

[3] Kern A, Perreten V 2013 Clinical and molecular features of methicillin-resistant, coagulase-negative staphylococci of pets and horses. J Antimicrob Chemother 68:1256–1266. doi:10.1093/jac/dkt020. 23425780

[4] Shah MM, Iihara H, Noda M, Song SX, Nhung PH, Ohkusu K, Kawamura Y, Ezaki T 2007 *dnaJ* gene sequence-based assay for species identification and phylogenetic grouping in the genus *Staphylococcus*. Int J Syst Evol Microbiol 57:25–30. doi:10.1099/ijs.0.64205-0. 17220435

[5] Kwok AY, Su SC, Reynolds RP, Bay SJ, Av-Gay Y, Dovichi NJ, Chow AW 1999 Species identification and phylogenetic relationships based on partial HSP60 gene sequences within the genus *Staphylococcus*. Int J Syst Bacteriol 49:1181–1192. doi:10.1099/00207713-49-3-1181. 10425778

[6] Drancourt M, Raoult D 2002 *rpoB* gene sequence-based identification of *Staphylococcus* species. J Clin Microbiol 40:1333–1338. doi:10.1128/JCM.40.4.1333-1338.2002. 11923353 PMC140360

[7] Mellmann A, Becker K, von Eiff C, Keckevoet U, Schumann P, Harmsen D 2006 Sequencing and staphylococci identification. Emerg Infect Dis 12:333–336. doi:10.3201/eid1202.050962. 16494767 PMC3373113

[8] Heikens E, Fleer A, Paauw A, Florijn A, Fluit AC 2005 Comparison of genotypic and phenotypic methods for species-level identification of clinical isolates of coagulase-negative staphylococci. J Clin Microbiol 43:2286–2290. doi:10.1128/JCM.43.5.2286-2290.2005. 15872257 PMC1153770

[9] Zankari E, Hasman H, Cosentino S, Vestergaard M, Rasmussen S, Lund O, Aarestrup FM, Larsen MV 2012 Identification of acquired antimicrobial resistance genes. J Antimicrob Chemother 67:2640–2644. doi:10.1093/jac/dks261. 22782487 PMC3468078

[10] Chen HJ, Hung WC, Lin YT, Tsai JC, Chiu HC, Hsueh PR, Teng LJ 2015 A novel fusidic acid resistance determinant, *fusF*, in *Staphylococcus cohnii*. J Antimicrob Chemother 70:416–419. doi:10.1093/jac/dku408. 25313205

